# Association of Low Arousal Threshold Obstructive Sleep Apnea Manifestations with Body Fat and Water Distribution

**DOI:** 10.3390/life13051218

**Published:** 2023-05-19

**Authors:** Wen-Hua Hsu, Cheng-Chang Yang, Cheng-Yu Tsai, Arnab Majumdar, Kang-Yun Lee, Po-Hao Feng, Chien-Hua Tseng, Kuan-Yuan Chen, Jiunn-Horng Kang, Hsin-Chien Lee, Cheng-Jung Wu, Yi-Chun Kuan, Wen-Te Liu

**Affiliations:** 1 School of Respiratory Therapy, College of Medicine, Taipei Medical University, Taipei 110301, Taiwan; b117105061@tmu.edu.tw; 2 Department of Neurology, Taipei Medical University-Shuang Ho Hospital, New Taipei City 235041, Taiwan; junioryang@tmu.edu.tw; 3 Brain and Consciousness Research Center, Taipei Medical University-Shuang Ho Hospital, New Taipei City 235041, Taiwan; 4 International Ph.D. Program in Gerontology and Long-Term Care, College of Nursing, Taipei Medical University, Taipei 110301, Taiwan; 5 Department of Civil and Environmental Engineering, Imperial College London, London SW7 2AZ, UK; ct619@imperial.ac.uk (C.-Y.T.); a.majumdar@imperial.ac.uk (A.M.); 6 Division of Pulmonary Medicine, Department of Internal Medicine, Taipei Medical University-Shuang Ho Hospital, New Taipei City 235041, Taiwan; leekangyun@tmu.edu.tw (K.-Y.L.); pohao@tmu.edu.tw (P.-H.F.); chtseng0925@tmu.edu.tw (C.-H.T.); 14388@s.tmu.edu.tw (K.-Y.C.); 7 Research Center of Artificial Intelligence in Medicine, Taipei Medical University, Taipei 110301, Taiwan; jhk@tmu.edu.tw; 8 Graduate Institute of Nanomedicine and Medical Engineering, College of Biomedical Engineering, Taipei Medical University, Taipei 110301, Taiwan; 9 Department of Psychiatry, Taipei Medical University Hospital, Taipei 110301, Taiwan; 10 Department of Otolaryngology, Taipei Medical University-Shuang Ho Hospital, New Taipei City 235041, Taiwan; 11 Department of Neurology, School of Medicine, College of Medicine, Taipei Medical University, Taipei 110301, Taiwan; 12 Taipei Neuroscience Institute, Taipei Medical University, Taipei 110301, Taiwan; 13 Dementia Center, Taipei Medical University-Shuang Ho Hospital, New Taipei City 235041, Taiwan; 14 Sleep Center, Taipei Medical University-Shuang Ho Hospital, New Taipei City 235041, Taiwan

**Keywords:** low arousal threshold, obstructive sleep apnea, visceral fat, trunk-to-limb fat ratio, extra-to-intracellular water ratio

## Abstract

Obstructive sleep apnea (OSA) with a low arousal threshold (low-ArTH) phenotype can cause minor respiratory events that exacerbate sleep fragmentation. Although anthropometric features may affect the risk of low-ArTH OSA, the associations and underlying mechanisms require further investigation. This study investigated the relationships of body fat and water distribution with polysomnography parameters by using data from a sleep center database. The derived data were classified as those for low-ArTH in accordance with criteria that considered oximetry and the frequency and type fraction of respiratory events and analyzed using mean comparison and regression approaches. The low-ArTH group members (*n* = 1850) were significantly older and had a higher visceral fat level, body fat percentage, trunk-to-limb fat ratio, and extracellular-to-intracellular (E–I) water ratio compared with the non-OSA group members (*n* = 368). Significant associations of body fat percentage (odds ratio [OR]: 1.58, 95% confident interval [CI]: 1.08 to 2.3, *p* < 0.05), trunk-to-limb fat ratio (OR: 1.22, 95% CI: 1.04 to 1.43, *p* < 0.05), and E–I water ratio (OR: 1.32, 95% CI: 1.08 to 1.62, *p* < 0.01) with the risk of low-ArTH OSA were noted after adjustments for sex, age, and body mass index. These observations suggest that increased truncal adiposity and extracellular water are associated with a higher risk of low-ArTH OSA.

## 1. Introduction

Obstructive sleep apnea (OSA) is a common sleep-related breathing disorder affecting an estimated one billion individuals worldwide [1]. It is characterized by partial or complete upper airway obstruction during sleep, leading to decreased oxygen delivery [2], thereby triggering arousal from sleep for the restoration of the upper airway muscle tone [3]. However, some patients have a higher propensity to be awakened prematurely in response to breathing disturbances; this condition is clinically defined as low arousal threshold (low-ArTH) OSA [4]. These patients may experience more frequent awakenings during the night, leading to lower sleep quality compared with patients with normal arousal threshold OSA. Known risk factors for low-ArTH OSA include aging [5] and obesity [6]; however, the impact of the body composition on the severity, manifestations, and development of low-ArTH OSA remains unclear.

OSA is typically diagnosed using polysomnography (PSG), which is a comprehensive examination that monitors various physiological parameters during sleep to determine the relevant indices, such as the apnea–hypopnea index (AHI), oxygen desaturation index (ODI), and arousal index (ArI) [7]. However, the arousal threshold is not easily measurable in patients with low-ArTH OSA, and alternatives, such as oxygen saturation levels and AHI, have been proposed to identify this OSA phenotype [8]. OSA-associated manifestations, namely oxygen desaturation, arousal response, and AHI, are also associated with anthropometric profiles. For instance, a study reported that body mass index (BMI) is an essential indicator of the risk of OSA severity [9]. Studies have observed that individuals with severe OSA had significantly higher mean BMI and a larger mean neck circumference than those without OSA (BMI = 34.55 vs. 29.83 kg/m^2^, *p* = 0.021; neck circumference = 40.84 vs. 36.11 cm, both *p* < 0.001) [10]. Another study used oximetry parameters during sleep and body profiles to differentiate between high- or low-ArTH OSA and insomnia. That study observed that patients with both high- and low-ArTH OSA had higher BMI and wider neck circumference and waist circumference compared with patients with insomnia [11]. 

A review indicated that obesity increased breathing work and interfered with ventilatory drive. Specifically, obesity was associated with a prolonged duration of oxygen desaturation during sleep, which can lead to sustained nocturnal hypoxemia and affect the arousal threshold [12]. Hypoxia has been identified to act as a neurocognitive depressant by impairing the synthesis and turnover mechanisms of various neurotransmitters in both human studies and animal models, and these interactions are associated with arousal threshold alteration [13,14]. A related study indicated that an increase in pharyngeal resistance during sleep due to aging may be related to a decreased arousal threshold [5]. Another study investigated the effects of certain medications (e.g., Acetazolamide) on electrolytes in body fluids, as well as on the respiratory system loop gain and arousal threshold [15]. However, mechanisms underlying the relationship between the body profile and low-ArTH OSA risk remain unclear, and no study has thoroughly investigated the effects of aspects of body composition, such as body fat and water distribution, on low-ArTH OSA.

Arousal from sleep can be triggered by several factors, such as respiratory events causing a decline in oxygen levels and augmenting the carotid body response [16]. Increased carbon dioxide levels can increase central nervous system receptor sensitivity, resulting in arousal [17]. As a respiratory event progresses, the ventilation effort and the respiratory system loop gain may progressively increase in response to blood gas level changes. However, these responses are associated with negative airway pressure, which can cause arousal [18]. Thus, body composition can directly or indirectly enhance arousal or increase the risk of low-ArTH OSA. Excess body fat is associated with the active inflammatory response associated with the restricted airway anatomy and elevated oxidative stress [19,20]. These physiological reactions may cause a high oxygen demand or limited airflow during sleep and may thereby interfere with the sleep–wake cycle by triggering arousal from sleep.

Excess extracellular water (ECW) is associated with aggravated sleep-disordered breathing, which may induce arousal [21]. A high arousal frequency was observed in patients with chronic renal failure undergoing dialysis for removing body water, which is a relatively low-effectiveness method [22]. Moreover, increased body water levels may lead to fluid accumulation in the pulmonary system or upper airways, resulting in edema and further exacerbating airway obstruction [23]. Although several mechanisms have been identified as aggravating OSA severity and triggering arousal from sleep, the associations between parameters related to body fat or water and the manifestations or risk of low-ArTH OSA remain underexplored.

This retrospective study investigated the potential associations between body composition indices, such as visceral fat level, body fat percentage, and body water distribution, and the risk of low-ArTH OSA in healthy individuals and patients with low-ArTH OSA who underwent PSG in a sleep center in Taiwan. We hypothesized that OSA risk and severity are influenced by body fat and water distribution, leading to increased AHI, ODI, and ArI. By using regression models, this study examined the effects of body composition on the presence and manifestations of low-ArTH OSA. Furthermore, this study elucidated mechanisms underlying the relationship between alterations in body composition and the presence and manifestations of low-ArTH OSA. This study also identified strategies to reduce the risk of developing or worsening low-ArTH OSA through these mechanisms.

## 2. Materials and Methods

### 2.1. Ethics

This retrospective study was approved by the Taipei Medical University-Joint Institutional Review Board (TMU-JIRB Serial number: N202004048 and N202212067). All procedures pertaining to data assessment, deidentification, statistical analysis, and data storage and maintenance were performed in accordance with the approved protocol. 

### 2.2. Study Population 

This study was a retrospective analysis of patient data obtained from the PSG database of the sleep center at Shuang Ho Hospital (New Taipei City, Taiwan) from June 2019 to December 2021. The inclusion criteria were as follows: (1) having complete data on PSG parameters and a total recording time of >6 h, (2) being 18–85 years old, (3) not having undergone any invasive surgery or noninvasive treatment for OSA, (4) not having regular use of hypnotic or psychotropic medications, (5) not having a diagnosis of central nervous system disorders (e.g., brain tumor, stroke, or major head trauma), and (6) not having a diagnosis of pulmonary system disorders (e.g., chronic obstructive pulmonary disease or lung cancer). Next, the obtained data were deidentified, and based on suggested criteria, the study population was divided into two groups: the non-OSA group and low-ArTH OSA group [8,24]. Specifically, patients with an AHI of <5 events/h were included in the non-OSA group. Those who met any two of the following criteria were included in the low-ArTH group: (1) minimum SpO_2_ (min-SpO_2_) > 82.5%, (2) AHI < 30 events/h, and (3) fraction of hypopnea (F-hypopnea = hypopnea index/AHI) > 58.3%. For example, although patients with severe OSA had a high AHI (≥30 events/h), they were still included in the low-ArTH group if most of their respiratory events were hypopnea (>58.3%) and if their minimum SpO_2_ was >82.5%. Data obtained from patients with OSA who did not meet the low-ArTH criteria were stratified into a separate group (non-low-ArTH OSA group), which was used for supplementary analysis.

### 2.3. Body Composition Variables 

The body composition variables were obtained from the aforementioned database of the sleep center. Prior to PSG, these variables were determined by measuring bioelectrical impedance with the Tanita MC-780 system (Tanita, Tokyo, Japan). First, the individuals were required to fast for at least 3 h and empty their bladders before the data were recorded. During data collection, they were instructed to stand still with their feet shoulder-width apart and to hold the induction metal handles with both arms straight down. The measurement system automatically obtained the visceral fat level and fat distribution, muscle tone, and water percentage from the whole-body scale and local scale (i.e., trunk and limbs or extracellular and intracellular). Next, we calculated indices concerning fat and water distribution: the ECW to intracellular water (ICW) ratio (ECW/ICW, E–I ratio) and the trunk-to-limb fat ratio (trunk fat mass/limb fat mass). 

### 2.4. PSG Parameters 

PSG was conducted at the sleep center by using three recording systems—Embla N7000 (ResMed, San Diego, CA, USA), Embletta MPR (Natus Medical, Pleasanton, CA, USA), and Nox-A1 (Nox Medical, Alpharetta, GA, USA)—and two scoring interfaces: RemLogic software (version 3.41; Embla Systems, Thornton, CO, USA) and Noxturnal system (version 6.2.2; Nox Medical). The physiological signals obtained in PSG were scored by a licensed PSG technologist in accordance with the 2017 American Academy of Sleep Medicine manual [25]. First, sleep architecture was identified using specific criteria based on brain wave activity, eye movements, and muscle tone, which included wake, non–rapid eye movement stages (N1, N2, and N3), and rapid eye movement stages. For the associated arousal events, PSG technologists scored intervals that contained the altered brainwave signals (≥3 s), namely high-frequency patterns that did not spindle (alpha wave: 8–12 Hz, theta wave: 4–8 Hz, high frequency: >16 Hz), and high-frequency patterns preceded by stable sleep (≥10 s), as arousal events. Pertaining to the respiratory event, apnea (≥90% reduction in oro-nasal thermistor signals), hypopnea (≥30% reduction in nasal prong pressure signals combined with ≥3% oxygen desaturation or arousal occurrence), and frequency of oxygen desaturation (≥3% oxygen desaturation) were scored. ArI, AHI, and ODI were then calculated based on the total event number divided by the total sleep time. OSA severity was categorized as none (AHI < 5 events/h), mild (AHI = 5–15 events/h), moderate (AHI = 15–30 events/h), and severe (AHI ≥ 30 events/h) [26]. Moreover, another technologist independently examined the scoring outcomes to reduce individual scoring bias, and variations in scoring were discussed to reach a consensus. 

### 2.5. Statistical Analysis 

SPSS (version 20.0; IBM, Armonk, NY, USA) was used for all statistical analyses. We employed two types of statistical regression models. First, multiple linear regression models were used to investigate the associations between body composition variables and PSG parameters. Both crude and adjusted models (adjusted for age, sex, and BMI) were established. Next, multivariable logistic regression models were used to explore the effect of body composition variables on enhancing the risk of low-ArTH OSA. The data of both groups were imputed into the two models. The odds ratio (OR), which indicates the association between body composition variables (i.e., visceral fat level, body fat percentage, trunk-to-limb fat ratio, and E–I water ratio) and the incidence of low-ArTH, was calculated. The level of significance was set at *p* < 0.05. 

## 3. Results

### 3.1. Basic Characteristics of Study Participants

The clinicodemographic data of the study participants stratified by the presence or absence of low-ArTH OSA are presented in Table 1. We included 2218 participants (368 in the non-OSA group and 1850 in the low-ArTH OSA group, with 745, 805, and 300 having mild, moderate, and severe OSA, respectively). Significant between-group differences were observed in age, BMI, and neck and waist circumferences. The mean visceral fat level, body fat percentage, trunk–limb fat distribution, and basal metabolic rate were significantly higher in the low-ArTH group than in the non-OSA group (*p* < 0.01). Compared with the non-OSA group, the low-ArTH OSA group exhibited a significantly higher mean total body water percentage and ECW percentage and a significantly lower mean ICW percentage. Correspondingly, the low-ArTH group exhibited a significantly higher mean E–I water distribution (0.73 ± 0.08) than the non-OSA group. Overall, compared with the non-OSA group, the low-ArTH OSA group had more visceral fat and body fat, especially in the trunk area, and a greater proportion of water in the extracellular space. 

### 3.2. PSG Variables of Study Participants

Table 2 provides between-group comparisons of PSG variables, including sleep architecture, and variables of the associated events (arousal and respiratory events). The low-ArTH OSA group exhibited similar NREM and REM stage distribution to the non-OSA group but had a significantly higher wake after sleep onset (WASO) time compared with the non-OSA group. The low-ArTH group demonstrated a significant extended duration in both apnea (non-OSA: 7.04 ± 9.42 s; low-ArTH: 14.36 ± 10.12 s) and hypopnea (non-OSA: 24.16 ± 7.56 s; low-ArTH: 26.13 ± 6.01 s). Additionally, AHI, ODI, and ArI were significantly higher in the low-ArTH group than in the non-OSA group.

### 3.3. Associations between PSG Variables, Visceral Fat Level, and Distribution of Body Fat and Body Water 

Table 3, Table 4 and Table 5 summarize the associations between PSG parameters and body composition measures, including visceral fat level (Table 3), trunk-to-limb fat ratio (Table 4), and E–I water ratio (Table 5). We observed that after adjustment for age, sex, and BMI, a 1-unit increase in the standard deviation of the fat level was significantly associated with a 3.78 events/h increase in the AHI (95% confidence interval [CI]: 1.75–5.81, *p* < 0.01), a 2.52 events/h increase in the ODI (95% CI: 0.8–4.25, *p* < 0.01), and a 2.96 events/h increase in the ArI (95% CI: 1.33–4.59, *p* < 0.01). Similarly, regarding the effect of the body fat distribution, a 1-unit increase in the trunk-to-limb fat ratio was significantly associated with increased AHI (β = 3.43, 95% CI: 0.53–6.33, *p* < 0.01) and ArI (β = 5.17, 95% CI: 2.85–7.49, *p* < 0.01) after adjustment for age, sex, and BMI. Next, regarding the effect of the body water distribution, a 1-unit increase in the E–I water ratio was significantly associated with increased WASO (β = 37.03, 95% CI: 8.83–65.24, *p* < 0.05) and ArI (β = 22.09, 95% CI: 14.89–29.29, *p* < 0.01) as well as decreased sleep efficiency (β = −24.73, 95% CI: −34.25 to −15.2, *p* < 0.01) and hypopnea duration (β = −4.99, 95% CI: −9.4 to −0.58, *p* < 0.05). 

### 3.4. Alteration in Body Fat and Water Associated with the Risk of Low-ArTH 

Table 6 presents the results of the logistic regression models examining the associations of parameters regarding body fat and body water between the two groups. In the crude models, significant associations were observed between an increment in the visceral fat level, body fat percentage, trunk-to-limb fat ratio, and E–I water ratio and the risk of low-ArTH OSA. Next, after adjustment for age, sex, and BMI, a 1% increase in body fat was significantly associated with a higher OR (1.58, 95% CI: 1.08–2.3, *p* < 0.05) of low-ArTH OSA, a 1-unit increment in the trunk-to-limb fat ratio was significantly associated with a higher OR (1.22, 95% CI: 1.04–1.43, *p* < 0.05) of low-ArTH OSA, and a 1-unit increment in the E–I water ratio was significantly associated with an increased OR (1.32, 95% CI: 1.08–1.62) of low-ArTH OSA.

### 3.5. Supplementary Analysis

The clinicodemographic and PSG data of the non-low-ArTH OSA group are illustrated in Table S1 (*n* = 706). The non-low-ArTH OSA group included 671 patients with severe OSA, accounting for 95.04% of the patients in the group. The mean AHI and ArI were 59.58 ± 22.8 and 32.1 ± 19.34 events/h, respectively. Associations between PSG parameters and body composition measures were determined, including the visceral fat level (Table S2), trunk-to-limb fat ratio (Table S3), and E–I water ratio (Table S4). After adjustment for age, sex, and BMI, we noted significant negative associations among the trunk-to-limb fat ratio, decreased hypopnea duration, AHI, and ODI in patients both with and without low-ArTH OSA. Similarly, concerning body water distribution, a 1-unit increase in the E–I water ratio was significantly associated with decreases in the apnea duration (β = −14.02, 95% CI: −20.97 to −7.08, *p* < 0.01) and hypopnea duration (β = −7.52, 95% CI: −11.54 to −3.5, *p* < 0.01) after adjustment for age, sex, and BMI. Table S5 lists the outcomes of logistic regression models investigating the relationship between body fat and body water parameters in patients with OSA with and without low ArTH. After adjustment for age, sex, and BMI, a 1-unit increase in the trunk-to-limb fat ratio was significantly associated with a higher odds ratio (1.18, 95% CI: 1.05–1.33, *p* < 0.01) of low-ArTH OSA, and a 1-unit increase in the E–I water ratio was significantly associated with an increased odds ratio (1.78, 95% CI: 1.52–2.09) of low-ArTH OSA.

## 4. Discussion

Anthropometric characteristics have been linked to the manifestations and severity of low-ArTH OSA. However, the effect of specific factors, such as visceral fat or body fat and water distribution, on this relationship has remained unexplored. Our retrospective study used data including PSG parameters and body composition variables, analyzed the association between these variables using regression models, and investigated the risk of low-ArTH OSA based on alterations in body composition variables.

OSA is believed to involve four endophenotypic traits, namely pharyngeal muscle responsiveness, respiratory drive instability (loop gain), arousal threshold, and anatomical factors [27]. These traits interact with each other and contribute to the periodic repetition of respiratory or frequent arousal events in patients with OSA. Recent studies have demonstrated that one of the endophenotypic traits, namely low-ArTH OSA, which may contribute to the highly frequent arousal response, may be associated with or even aggravate other related diseases (e.g., periodic limb movements, cognitive impairment, Alzheimer’s disease) [28,29,30]. Therefore, understanding the mechanisms underlying these endophenotypic OSA traits could help to prevent the disease presentation or allow for precise OSA treatment based on the specific pathophysiological condition of each individual. In light of these scenarios, this study investigated the relationship between body composition indices and the severity or manifestations of low-ArTH OSA. This study also explored whether alterations in body composition affect the presence of low-ArTH OSA.

Our data indicated that after adjustments for age, sex, and BMI, the visceral fat level was significantly associated with increased AHI, ODI, and ArI, whereas the trunk-to-limb fat ratio was significantly associated with AHI and ArI. Body fat accumulation, especially in the abdominal region, has been linked to the aggravated symptoms of sleep-disordered breathing, including high frequency of apnea, hypopnea or arousal, and severe oxygen desaturation. More precisely, excessive trunk and visceral adiposity may directly increase the pressure on the diaphragm, thereby increasing the work of breathing and causing difficulty in breathing during sleep [31]. This can also reduce lung capacity by limiting diaphragmatic movement [32]. High visceral adiposity can also increase the whole-body oxygen demand, thus elevating the risks of nocturnal hypoxia and arousal from sleep [33]. Furthermore, some researchers have observed an increase in the resistance of hormones such as leptin and insulin, as well as a reduction in melatonin levels [34]. These hormonal changes can directly affect the sleep–wake cycle and body weight control and can increase the frequency of arousal from sleep and nocturnal hypoxemia. Excess adiposity can increase inflammatory biomarkers and oxidative stress, leading to active systemic inflammation and increased oxygen demand and further worsening sleep-disordered breathing symptoms [35,36]. Anatomically, excess truncal fat mass indicates a significant accumulation of adiposity in the anterior chest wall, which may lead to reduced chest wall compliance and respiratory muscle endurance, as well as increased work of breathing and airway resistance [37]. Consistent with previous findings, we observed significant associations between the visceral fat level and trunk-to-limb fat ratio in the low-ArTH OSA group. A study reported that visceral fat was a predominant indicator in the evaluation of the AHI level and nocturnal hypoxemia severity [38]. Another study reported that excess visceral fat can cause severe hypoxemia during sleep and an increased risk of metabolic syndrome [39]. Taken together, these results indicate that excess visceral and trunk adiposity are associated with alterations in the anatomy, physical characteristics, and oxygen demand in the respiratory system. All of these physiological reactions may cause frequent arousal from sleep.

Regarding the effect of the trunk-to-limb fat ratio on alterations in the arousal threshold, the retrieved outcomes of the supplementary analysis (when focusing on data from patients with or without low-ArTH OSA) indicated that this parameter was significantly associated with decreased hypopnea duration, AHI, and ODI after adjustment for age, sex, and BMI. Previous studies have suggested that excess body adiposity is significantly associated with the risk of sustained hypoxia, which can interfere with neurochemical metabolism or transmission and affect the arousal threshold [40]. However, the effect of fat distribution on the arousal threshold with consideration of BMI should be comprehensively investigated. The present outcomes demonstrated that an increased trunk-to-limb fat ratio was associated with a decreased arousal threshold, and this finding may be accounted for by some potential underlying mechanisms. Excessive fat in the upper body trunk may increase airway resistance and thus result in a highly sensitive respiratory load, causing the patient to wake up in response to small increases in inspiratory effort [41]. The high trunk-to-limb fat ratio was associated with airway narrowing and epiglottic pressure alterations [42], which have been indicated as surrogates for measuring the arousal threshold [43]. Together, the retrieved results imply that excessive adiposity in the upper body trunk is associated with a change in the arousal threshold. 

With adjustments for age, sex, and BMI, our data indicated that the E–I water ratio was associated with reduced sleep efficiency and hypopnea duration and increased WASO and ArI. In the supplementary analysis, when focusing on data obtained from patients with or without low-ArTH OSA, the elevated E–I water ratio was associated with decreased sleep efficiency, increased WASO, and shortened apnea and hypopnea durations. These findings may imply that the E–I water ratio is associated with a decreased arousal threshold. In a supplementary analysis, when focusing on data obtained from patients with or without low-ArTH OSA, the elevated E–I water ratio was associated with decreased sleep efficiency and decreased apnea and hypopnea durations. This finding may imply that the E–I water ratio is associated with a decreased arousal threshold. Although the precise underlying mechanisms remain unclear, changes in fluid balance may contribute to sleep disturbances. First, excess ECW can affect the concentrations of extracellular electrolytes. Altered ion concentrations in interstitial brain tissues are associated with neural or cortical activity and the ascending arousal system, which can directly affect the sleep–wake cycle and sleep stages, potentially leading to more frequent awakenings or alterations in the sleep stage duration [44]. Regarding the effect of excess ECW on the tracts of the pulmonary system, the retention or accumulation of ECW may contribute to overnight fluid relocation from the lower body to the upper airway due to the supine position during sleep [45]. However, such redistributed fluids may cause airway swelling or complete obstruction, which can limit breathing airflow, cause arousal from sleep, and shorten the respiratory event duration [46]. Conversely, a study in which dehydration was performed through nocturnal peritoneal dialysis reported improved sleep quality with decreased AHI, decreased frequency of arousal from sleep, and reduced airway congestion [47]. Moreover, alterations in the body’s water distribution can cause changes in the partial pressure of carbon dioxide and oxygen in the blood, affecting the ventilatory drive and thereby inducing arousal from sleep in response to the respiratory events [48]. Studies have reported similar findings in line with the significant associations between ECW and low-ArTH OSA symptoms determined in this study. For example, a study found significantly higher AHI and ArI and lower sleep efficiency before dialysis than after dialysis was performed to remove extracellular fluid [49]. A systematic review concluded that excess ECW may cause a high vascular tone and deleterious sleep disturbances by interfering with the sleep–wake cycle [50]. Based on the determined associations between the E–I water ratio and low-ArTH OSA risk, the present results suggest that considering the body water distribution may be important for alleviating the symptoms of such sleep disorders.

The current study also indicated that increments in the body fat percentage, trunk-to-limb fat ratio, and E–I water were associated with an elevated risk of low-ArTH OSA. Several reasons can explain these observations. The arousal threshold can be manipulated pharmacologically by manipulating the interactions of the respiratory system or the central nervous system [51,52]. This threshold can be considered as the action point beyond which an arousal response is generated in response to the accumulated respiratory stimuli. However, respiratory and central nervous system abnormalities, such as imbalanced levels of oxygen and carbon dioxide leading to an out-of-range pH scale or frequent airway obstructions causing restricted airflow and enhanced ventilator drive loop gain, may impair the arousal threshold and cause minor respiratory events [53]. Therefore, excess body fat, particularly truncal adiposity, and increased ECW may lead to an increased number of respiratory events or an imbalanced risk of gas exchange related to the ventilator drive, resulting in an elevated risk of low-ArTH OSA.

Our study has some strengths. Approximately 30–50% of all patients with OSA have the low-ArTH phenotype, which is characterized by the instability of ventilatory control and a tendency to induce arousal from sleep in response to slight respiratory stimuli [54]. Despite this observation, current research has primarily focused on patient compliance with or the efficiency of low-ArTH OSA treatment [55,56]. To the best of our knowledge, our study is the first to comprehensively investigate the relationships between body composition and low-ArTH OSA. The findings suggest that body fat in the trunk or visceral fat was associated with oxygen desaturation and directly worsened the severity of low-ArTH OSA. Furthermore, the distribution of body water, especially ECW, can affect the arousal response or threshold and increase the frequency of arousal.

This study has certain limitations. First, this study enrolled participants from a single center in Taiwan, thereby limiting the generalizability of the findings. We exclusively considered body variables instead of craniofacial factors, commonly considered predictors of respiratory events or the occurrence of arousal from sleep [57]. Next, PSG, which determines the AHI, is the clinical standard for classifying the severity of OSA. However, the PSG results were scored by various technologists, and scoring variability may have affected the PSG examination outcomes [58]. Although we obtained the data from a sleep center that regularly conducted training to ensure interscore consistency, the results may still have been influenced by scoring variability. The first-night effect is a phenomenon that occurs on the first night of testing and is characterized by an altered sleep cycle and affected sleep physiology. This effect can cause inaccuracies in the PSG results [59]. Although we attempted to mitigate this effect by excluding the data of patients with sleep efficiency below 40%, PSG parameters may still have been partially affected, leading to some degree of bias. Finally, because OSA is a multifactorial sleep-disordered breathing condition, factors such as the presence of comorbidities, socioeconomic status, smoking or alcohol use, and menopausal status can considerably affect its severity [60,61]. Thus, the absence of information on comorbidities, habitual and other lifestyle factors, and other personal background details that may interact with OSA may have partially deterred the enhancement of our observations. Future studies should use questionnaire surveys to determine the aforementioned details and enhance the robustness of the derived associations. 

## 5. Conclusions

To understand the effects of body composition on the manifestation and incidence of low-ArTH OSA, we analyzed the PSG parameters and body composition data of patients from a sleep center in northern Taiwan and investigated the relationship between body composition variables and the risk of low-ArTH OSA. We observed that increased levels of visceral fat and the trunk-to-limb fat ratio were significantly associated with aggravated OSA severity and fragmented sleep, as evidenced by higher AHI or ArI. Excess ECW levels (high E–I water ratio) were significantly associated with increased ArI, reduced hypopnea duration, increased WASO, and decreased sleep efficiency. Moreover, increased body fat, particularly truncal adiposity, and an increased E–I water ratio were significantly associated with a higher risk of low-ArTH OSA. Altogether, the results revealed that body fat or visceral fat was related to oxygen desaturation and directly aggravated the severity of low-ArTH OSA, whereas body water distribution, especially ECW, affected the arousal response or threshold. Additionally, both excess body fat and ECW can increase the risk of low-ArTH OSA. Future interventions for preventing low-ArTH OSA must alleviate excess body fat and water. 

## Figures and Tables

**Table 1:** Anthropometric data of participants stratified according to the criteria of low arousal threshold OSA (Low-ArTH).

Categorical Variables	Non-OSA Group (*n* = 368)	Low-ArTH Group (*n* = 1850)	*p*
Age (years)	39.08 ± 12.43	48.83 ± 13.55	<0.01
Sex (men/women) *	132/236	1121/729	<0.01
BMI (kg/m^2^)	22.74 ± 3.53	25.99 ± 4.26	<0.01
Neck circumference (cm)	33.9 ± 4.1	36.88 ± 4.49	<0.01
Waist circumference (cm)	79.09 ± 10.39	89.82 ± 10.8	<0.01
Body composition			
Visceral fat level (score)	6.35 ± 3.9	10.95 ± 4.27	<0.01
Body Fat percent (%)	26.61 ± 8.0	28.61 ± 8.94	<0.01
trunk–limb fat ratio	1.17 ± 0.23	1.35 ± 0.2	<0.01
Muscle percent (%)	18.36 ± 4.02	17.79 ± 4.8	0.41
Basal metabolic rate (kcal)	5449.27 ± 982.4	6072.31 ± 1152.2	<0.01
Body water distribution			
TBW (%)	50.97 ± 4.93	49.74 ± 5.46	<0.01
ECW (%)	40.95 ± 2.03	41.96 ± 2.61	<0.01
ICW (%)	59.05 ± 2.03	58.04 ± 2.61	<0.01
E-I water ratio	0.7 ± 0.06	0.73 ± 0.08	<0.01
OSA severity (*n*, %) *			<0.01
Normal	368 (100%)	-	
Mild	-	745 (40.27%)	
Moderate	-	805 (43.51%)	
Severe	-	300 (16.22%)	

Definition of abbreviations: Low-ArTH—low arousal threshold; BMI—body mass index; trunk–limb fat distribution—distribution of trunk fat mass to limb fat mass; TBW—total body water; ICW—intracellular water; ECW—extracellular water; E-I water ratio—ratio of extracellular to intracellular water; OSA—obstructive sleep apnea. Data are expressed as mean ± standard deviation. Differences between groups were assessed using a Mann–Whitney U-test. * Differences between groups were assessed using a chi-square test. Note: Calculation of trunk–limb fat ratio: trunk fat (kg)/limb fat (kg); Calculation of TBW (%): TBW (kg)/body weight (kg) × 100; Calculation of ECW (%): ECW (kg)/TBW (kg) × 100; Calculation of ICW (%): ICW (kg)/ TBW (kg) × 100; Calculation of E–I ratio: ECW/ICW.

**Table 2:** Comparison of the PSG parameters between the non-OSA and the low-ArTH groups.

Categorical Variables	Non-OSA Group (*n* = 367)	Low-ArTH Group (*n* = 1784)	*p*
Sleep architecture			
Sleep efficiency (%)	77.55 ± 12.99	76.25 ± 13.28	0.08
NREM (% of TST)	85.65 ± 7.42	85.9 ± 7.03	0.66
REM (% of TST)	14.34 ± 7.42	14.1 ± 7.03	0.67
WASO (min)	47.85 ± 39.92	57.33 ± 40.34	<0.01
Event Duration (s)			
Apnea	7.04 ± 9.42	14.36 ± 10.12	<0.01
Hypopnea	24.16 ± 7.56	26.13 ± 6.01	<0.01
Low ArTH criteria			
AHI (events/h)	2.6 ± 1.27	20.56 ± 13.32	<0.01
minSpO_2_ (%)	92.58 ± 2.85	86.37 ± 5.53	<0.01
F-hypopnea (%)	92.51 ± 12.61	89.11 ± 13.6	<0.01
Sleep disorder variables (events/h)			
Oxygen desaturation index	1.17 ± 1.13	13.96 ± 12.01	<0.01
Arousal index	11.87 ± 7.14	17.79 ± 10.26	<0.01

Abbreviations: PSG—polysomnography; TST—total sleep time; NREM—non–rapid eye movement; REM—rapid eye movement; WASO—wake time after sleep onset; Low ArTH—low arousal threshold; AHI—apnea–hypopnea index; SpO_2_—oxygen saturation as measured using pulse oximetry. Data are expressed as mean ± standard deviation. Differences between groups were assessed using a Mann–Whitney U test.

**Table 3:** Associations between sleep parameters and visceral fat level.

Categorical Variables	β Coefficient (95% CI)	
	Crude Model ^a^	Adjusted Model ^b^
Sleep architecture		
Sleep efficiency (%)	−0.58 (−1.14 to −0.03) *	−2.14 (−4.3 to 0.01)
WASO (min)	4.83 (3.16 to 6.5) **	1.69 (−4.67 to 8.05)
Event Duration (s)		
Apnea	1.22 (0.79 to 1.64) **	−0.69 (−2.37 to 0.98)
Hypopnea	0.01 (−0.25 to 0.28)	0.13 (−0.86 to 1.12)
Low ArTH criteria		
AHI (events/h)	6.12 (5.6 to 6.64) **	3.78 (1.75 to 5.81) **
minSpO_2_ (%)	−1.96 (−2.18 to −1.74) **	0.65 (−0.22 to 1.52)
F-hypopnea (%)	−0.35 (−0.92 to 0.21)	−1.15 (−3.38 to 1.07)
Sleep disorder variables (events/h)		
Oxygen desaturation index	5.62 (5.18 to 6.06) **	2.52 (0.8 to 4.25) **
Arousal index	1.31 (0.89 to 1.72) **	2.96 (1.33 to 4.59) **

Definition of abbreviations: WASO—wake time after sleep onset; Low ArTH—low arousal threshold; AHI—apnea–hypopnea index; SpO_2_—Oxygen saturation as measured using pulse oximetry. Changes in outcomes are presented as a standard deviation increase in visceral fat. ^a^ Simple linear regression models. ^b^ Multivariable linear regression models adjusted for age, sex, and body mass index. * *p* < 0.05; ** *p* < 0.01.

**Table 4:** Associations between sleep parameters and trunk–limb fat ratio.

Categorical Variables	β Coefficient (95% CI)	
	Crude Model ^a^	Adjusted Model ^b^
Sleep architecture		
Sleep efficiency (%)	−4.47 (−7.02 to −1.92) **	−2.42 (−5.49 to 0.66)
WASO (min)	24.61 (16.88 to 32.34) **	4.11 (−4.96 to 13.18)
Event Duration (s)		
Apnea	6.43 (4.45 to 8.42) **	1.28 (−1.11 to 3.67)
Hypopnea	2.43 (1.21 to 3.64) **	−0.15 (−1.57 to 1.27)
Low ArTH criteria		
AHI (events/h)	18.18 (15.61 to 20.75) **	3.43 (0.53 to 6.33) **
minSpO_2_ (%)	−5.13 (−6.2 to −4.05) **	−0.39 (−1.63 to 0.85)
F-hypopnea (%)	−3.8 (−6.4 to −1.2) *	−1.73 (−4.9 to 1.44)
Sleep disorder variables (events/h)		
Oxygen desaturation index	13.87 (11.63 to 16.1) **	0.29 (−2.18 to 2.76)
Arousal index	7.35 (5.44 to 9.27) **	5.17 (2.85 to 7.49) **

Definition of abbreviations: trunk–limb fat ratio—distribution of trunk fat mass to limb fat mass; WASO—wake time after sleep onset; Low ArTH—low arousal threshold; AHI—apnea–hypopnea index; SpO_2_—Oxygen saturation as measured using pulse oximetry. ^a^ Simple linear regression models. ^b^ Multivariable linear regression models adjusted for age, sex, and body mass index. * *p* < 0.05; ** *p* < 0.01.

**Table 5:** Associations between sleep parameters and extracellular water to intracellular water.

Categorical Variables	β Coefficient (95% CI)	
	Crude Model ^a^	Adjusted Model ^b^
Sleep architecture		
Sleep efficiency (%)	−31.19 (−38.17 to −24.21) **	−24.73 (−34.25 to −15.2) **
WASO (min)	97.51 (76.22 to 118.81) **	37.03 (8.83 to 65.24) *
Event Duration (s)		
Apnea	−4.94 (−10.5 to 0.62)	−1.91 (−9.35 to 5.53)
Hypopnea	−9.37 (−12.74 to −5.99) **	−4.99 (−9.4 to −0.58) *
Low ArTH criteria		
AHI (events/h)	24.22 (16.84 to 31.6) **	8.49 (−0.56 to 17.54)
minSpO_2_ (%)	−6.69 (−9.72 to −3.66) **	1.03 (−2.83 to 4.89)
F-hypopnea (%)	5.21 (−2.03 to 12.44)	−3.06 (−12.93 to 6.81)
Sleep disorder variables (events/h)		
Oxygen desaturation index	16.77 (10.39 to 23.15) **	−0.78 (−8.47 to 6.91)
Arousal index	24.52 (19.23 to 29.82) **	22.09 (14.89 to 29.29) **

Definition of abbreviations: WASO—wake time after sleep onset; Low ArTH—low arousal threshold; AHI—apnea–hypopnea index; SpO_2_—Oxygen saturation as measured using pulse oximetry. ^a^ Simple linear regression models. ^b^ Multivariable linear regression models adjusted for age, sex, and body mass index. * *p* < 0.05; ** *p* < 0.01.

**Table 6:** Associations (odd ratios, ORs) of the anthropometric measurements between the non-OSA and the low-ArTH groups.

Arousal Variables (Arousals/h)	Crude OR (95% CI) ^a^	Adjusted OR (95% CI) ^b^
Body composition		
Visceral fat level (score)	3.68 (3.15 to 4.31) **	1.11 (0.66 to 1.87)
Body fat percentage (%)	1.27 (1.13 to 1.42) **	1.58 (1.08 to 2.30) *
Trunk–limb fat ratio	2.36 (2.08 to 2.67) **	1.22 (1.04 to 1.43) *
Body water distribution		
E-I water ratio	1.59 (1.39 to 1.81) **	1.32 (1.08 to 1.62) **

Definition of abbreviations: Low ArTH—low arousal threshold; CI—confidence interval; trunk–limb fat ratio—ratio of trunk fat mass to limb fat mass; E-I water ratio—ratio of extracellular to intracellular water. ^a^ Simple logistic regression models. ^b^ Multivariable logistic regression models adjusted for age, sex, and body mass index. * *p* < 0.05; ** *p* < 0.01.

## Data Availability

Data for this study were retrospectively retrieved between June 2019 and December 2021 at the Sleep Center of Taipei Medical University-Shuang Ho Hospital. As our data contain personal information, these have not been provided in the supplement file. Interested parties may contact the corresponding author for access to the data set and relevant documents.

## Supplementary Materials

The following supporting information can be downloaded from https://www.mdpi.com/article/10.3390/life13051218/s1, Table S1: Anthropometric and polysomnography data of patients with obstructive sleep apnea without a low arousal threshold; Table S2: Associations between sleep parameters and visceral fat level in patients with or without low-ArTH OSA; Table S3: Associations between sleep parameters and trunk–limb fat ratio in patients with or without low-ArTH OSA; Table S4: Associations between sleep parameters and the extracellular water to intracellular water ratio in patients with or without low-ArTH OSA; Table S5: Associations (odds ratios, ORs) of anthropometric measurements between patients with OSA with or without low-ArTH.
