# Supplementary material for: Association of Low Arousal Threshold Obstructive Sleep Apnea Manifestations with Body Fat and Water Distribution
Source: Life (Basel). 2023 May 19;13(5):1218. doi: 10.3390/life13051218 (PMC10223607; doi:10.3390/life13051218)
Supplement: Supplementary file 1 [file life-13-01218-s001.zip › life-2322229-supplementary.pdf]

Supplementary Data

Table S1. Anthropometric and polysomnography data of patients with obstructive sleep apnea without a low arousal threshold (n = 706).

Categorical variables		Categorical variables	
Age (years)	46.29 ± 11.5	**Sleep architecture**	
Sex (male/female) *	656/50	Sleep efficiency (%)	77.13 ± 13.09
BMI (kg/m^2)	30.05 ± 4.93	NREM (% of TST)	86.75 ± 6.73
Neck circumference (cm)	40.83 ± 4.55	REM (% of TST)	13.25 ± 6.73
Waist circumference (cm)	100.94 ± 11.77	WASO (min)	60.95 ± 42.12
**Body composition**		**Event Duration** (s)	
Visceral fat level (score)	15.50 ± 4.28	Apnea	21.79 ± 7.61
Body fat percentage (%)	29.40 ± 6.45	Hypopnea	25.54 ± 6.18
Trunk/limb fat ratio	1.37 ± 0.14	**Low ArTH criteria**	
Muscle percentage (%)	18.53 ± 3.44	AHI (events/h)	59.58 ± 22.8
Basal metabolic rate (kcal)	7269.68 ± 990.69	minSaO2 (%)	73.52 ± 7.84
**Body water distribution**		F-hypopnea (%)	60.48 ± 27.89
TBW (%)	48.38 ± 4.66	**Sleep disorder variables** (events/h)	
ECW (%)	40.56 ± 1.22	Oxygen desaturation index	54.20 ± 23.97
ICW (%)	59.44 ± 1.22	Arousal index	32.10 ± 19.34
E–I water ratio	0.68 ± 0.03		
**OSA severity** (*n*, %) *			
Mild	8 (1.13%)		
Moderate	27 (3.82%)		
Severe	671 (95.04%)		

Definition of abbreviations—Low-ArTH: low arousal threshold; BMI: body mass index; trunk/limb fat distribution: the distribution of trunk fat mass to limb fat mass; TBW: total body water; ICW: intracellular water; ECW: extracellular water; E–I water ratio: the distribution of extracellular to intracellular water; OSA: obstructive sleep apnea; PSG: polysomnography; TST: total sleep time; NREM: non–rapid eye movement; REM: rapid eye movement; WASO: wake time after sleep onset; AHI: apnea–hypopnea index; SpO_2: oxygen saturation as measured using pulse oximetry.

Data are expressed as the mean ± standard deviation.

Note: Calculation of trunk/limb fat ratio: trunk fat (kg) / limb fat (kg); Calculation of TBW (%): TBW (kg) / body weight (kg) × 100; Calculation of ICW (%): ICW (kg) / TBW (kg) × 100; Calculation of ECW (%): ECW (kg) / TBW (kg) × 100; Calculation of E–I ratio: ECW / ICW.

Table S2. Associations between sleep parameters and visceral fat level in patients with or without low-ArTH OSA (1850 patients with low-ArTH and 706 patients without low-ArTH).

Categorical variables	β coefficient (95% CI)	
	Crude model [a]	Adjusted model [b]
**Sleep architecture**		
Sleep efficiency (%)	−0.90 (−1.41 to −0.39) **	−2.39 (−4.61 to −0.18) *
WASO (min)	5.44 (3.87 to 7.01) **	1.50 (−5.13 to 8.13)
**Event Duration** (s)		
Apnea	1.31 (0.92 to 1.69) **	0.34 (−1.32 to 2.0)
Hypopnea	−0.69 (−0.92 to −0.45) **	−0.19 (−1.15 to 0.77)
**Low ArTH criteria**		
AHI (events/h)	12.69 (11.9 to 13.48) **	2.03 (−1.42 to 5.47)
minSpO_2 (%)	−3.79 (−4.08 to −3.49) **	0.73 (−0.55 to 2.01)
F-hypopnea (%)	−5.15 (−6.01 to −4.3) **	−2.65 (−6.37 to 1.07)
**Sleep disorder variables** (events/h)		
Oxygen desaturation index	13.23 (12.44 to 14.02) **	0.11 (−3.29 to 3.52)
Arousal index	4.37 (3.82 to 4.92) **	3.37 (0.95 to 5.79) *

Definition of abbreviations—WASO: wake time after sleep onset; Low-ArTH: low arousal threshold; AHI: apnea–hypopnea index; SpO_2: Oxygen saturation as measured using pulse oximetry.

Changes in outcomes are described in terms of a standard deviation increase in the visceral fat level.

[a] Simple linear regression models.

[b] Multivariable linear regression models were adjusted for age, sex, and body mass index.

*P < .05; **P < .01.

Table S3. Associations between sleep parameters and trunk/limb fat ratio in patients with or without low-ArTH OSA (1850 patients with low-ArTH and 706 patients without low-ArTH).

Categorical variables	β coefficient (95% CI)	
	Crude model [a]	Adjusted model [b]
**Sleep architecture**		
Sleep efficiency (%)	−5.18 (−7.95 to −2.42) **	−3.10 (−6.12 to −0.08) *
WASO (min)	21.11 (12.58 to 29.64) **	2.97 (−6.07 to 12.02)
**Event Duration** (s)		
Apnea	4.64 (2.54 to 6.74) **	−1.29 (−3.56 to 0.97)
Hypopnea	1.53 (0.26 to 2.8) *	−1.57 (−2.88 to -0.26) *
**Low ArTH criteria**		
AHI (events/h)	10.92 (5.91 to 15.93) **	−8.30 (−12.99 to −3.61) **
minSpO_2 (%)	−2.32 (−4.1 to −0.54) *	3.20 (1.46 to 4.95) **
F-hypopnea (%)	−5.95 (−10.69 to −1.21) *	5.79 (0.72 to 10.86) *
**Sleep disorder variables** (events/h)		
Oxygen desaturation index	8.80 (3.73 to 13.86) **	−10.58 (−15.21 to −5.95) **
Arousal index	5.61 (2.5 to 8.71) **	−0.96 (−4.26 to 2.35)

Definition of abbreviations—WASO: wake time after sleep onset; Low-ArTH: low arousal threshold; AHI: apnea–hypopnea index; SpO_2: Oxygen saturation as measured using pulse oximetry.

[a] Simple linear regression models.

[b] Multivariable linear regression models adjusted for age, sex, and body mass index.

*P < .05; **P < .01.

Table S4. Associations between sleep parameters and the extracellular water to intracellular water ratio in patients with or without low-ArTH OSA (1850 patients with low-ArTH and 706 patients without low-ArTH).

**Categorical variables**	**β coefficient (95% CI)**	
	**Crude model [a]**	**Adjusted model [b]**
**Sleep architecture**		
Sleep efficiency (%)	−30.55 (−37.48 to −23.63) **	−24.89 (−34.14 to −15.65) **
WASO (min)	83.11 (61.63 to 104.59) **	34.91 (7.14 to 62.68) *
**Event Duration** (s)		
Apnea	−21.45 (−26.73 to −16.17) **	−14.02 (−20.97 to −7.08) **
Hypopnea	−8.40 (−11.6 to −5.19) **	−7.52 (−11.54 to −3.5) **
**Low ArTH criteria**		
AHI (events/h)	−55.59 (−68.17 to −43.02) **	−37.16 (−51.54 to −22.78) **
minSpO_2 (%)	20.17 (15.73 to 24.62) **	16.50 (11.17 to 21.84) **
F-hypopnea (%)	58.2 (46.38 to 70.02) **	31.18 (15.63 to 46.73) **
**Sleep disorder variables** (events/h)		
Oxygen desaturation index	−61.93 (−74.58 to −49.28) **	−45.20 (−59.38 to −31.03) **
Arousal index	−4.86 (−12.75 to 3.02) **	0.86 (−9.31 to 11.03)

Definition of abbreviations—WASO: wake time after sleep onset; Low-ArTH: low arousal threshold; AHI: apnea–hypopnea index; SpO_2: Oxygen saturation as measured using pulse oximetry.

[a] Simple linear regression models.

[b] Multivariable linear regression models adjusted for age, sex, and body mass index.

*P < .05; **P < .01.

Table S5. Associations (odds ratios, ORs) of anthropometric measurements between patients with OSA with or without low-ArTH

Arousal variables (arousals/h)	Crude OR (95% CI) [a]	Adjusted OR (95% CI) [b]
**Body composition**		
Visceral fat level (score)	0.31 (0.28 to 0.35) **	1.07 (0.67 to 1.72)
Body fat percentage (%)	0.91 (0.83 to 0.99) *	1.28 (0.96 to 1.72)
Trunk/limb fat ratio	0.90 (0.83 to 0.98) *	1.18 (1.05 to 1.33) **
**Body water distribution**		
E–I water ratio	2.18 (1.94 to 2.45) **	1.78 (1.52 to 2.09) **

Definition of abbreviations—Low-ArTH: low arousal threshold; CI: confidence interval; trunk/limb fat ratio: ratio of trunk fat mass to limb fat mass; E–I water ratio: ratio of extracellular to intracellular water.

[a] Simple logistic regression models.

[b] Multivariable logistic regression models adjusted for age, sex, and body mass index.

*P < .05; **P < .01.
